# Acanthomatous Ameloblastoma of Mandible in a Paediatric Patient

**DOI:** 10.1155/2018/6594675

**Published:** 2018-10-21

**Authors:** Narasimhan Malathi, G. V. V. Giri, Deepak A. Pandyan, Ramalingam Suganya, Harikrishnan Thamizhchelvan

**Affiliations:** ^1^Professor & Head, Department of Oral Pathology, Faculty of Dental Sciences, Sri Ramachandra Institute of Higher Education & Research (DU), Porur, Chennai, India; ^2^Professor, Department of Oral & Maxillofacial Surgery, Faculty of Dental Sciences, Sri Ramachandra Institute of Higher Education & Research (DU), Porur, Chennai, India; ^3^Assoc. Professor, Department of Oral &Maxillofacial Surgery, Faculty of Dental Sciences, Sri Ramachandra Institute of Higher Education & Research (DU), Porur, Chennai, India; ^4^Senior Lecturer in Oral Pathology, Department of Oral &Maxillofacial Surgery, Faculty of Dental Sciences, Sri Ramachandra Institute of Higher Education & Research (DU), Porur, Chennai, India; ^5^Professor, Department of Oral Pathology, Faculty of Dental Sciences, Sri Ramachandra Institute of Higher Education & Research (DU), Porur, Chennai, India

## Abstract

Ameloblastoma is a slow-growing, benign odontogenic tumor derived from odontogenic epithelial components with a mature fibrous stroma. It is the second most common odontogenic neoplasm following odontome. Acanthomatous ameloblastoma histologically presents with squamous epithelial metaplastic transformation of odontogenic tissue. The present case report of a 12-year-old male exclusively elaborates the issues concerned with the aggressive nature of acanthomatous ameloblastoma (AA) which is a distinctive variant of ameloblastoma.

## 1. Introduction

The word ameloblastoma is derived from a combination of the French word “Amel” meaning enamel and the Greek word “Blastos” which means germ or bud. Ameloblastomas are locally invasive, slow-growing odontogenic tumors most commonly involving the mandible. Based on its size, anatomical location, histological variant, and involvement of lesion in adjacent anatomical areas, there are varying treatment modalities available for these tumors [1]. In paediatric population, the occurrence of ameloblastomas is about 10%–15% [2].

## 2. Case Report

**Figure 1:**
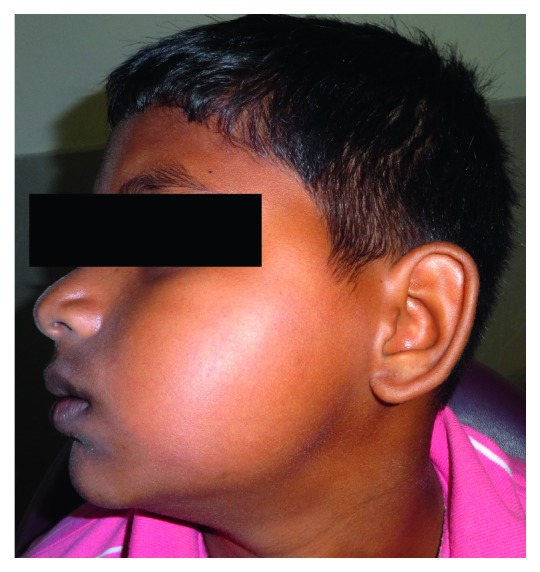
Clinical photograph showing diffuse swelling on the left side of the face.

A 12-year-old boy reported to our institute with a chief complaint of swelling on the left side of the face for the past six months (Figure 1). The patient was apparently normal 6 months back after which he noticed the swelling. The patient's general, family, and medical history was not contributory.

Extraorally, there was a presence of diffuse swelling involving the left side of the face, extending superiorly to the left zygomatic arch, inferiorly to the lower border of the mandible, anteriorly to the corner of the mouth, and posteriorly to the retromolar portion of the mandible. There was no obliteration of nasolabial folds seen. On palpation, the swelling was hard, noncompressible, and nonreducible along with diffuse expansion of the middle and lower 3rd of the facial region.

Intraoral examination revealed a swelling involving the retromolar trigone and molar regions with obliteration of the mucobuccal fold. On palpation, the lesion was firm to hard in consistency and nontender with expansion of left buccolingual cortical plates.

**Figure 2:**
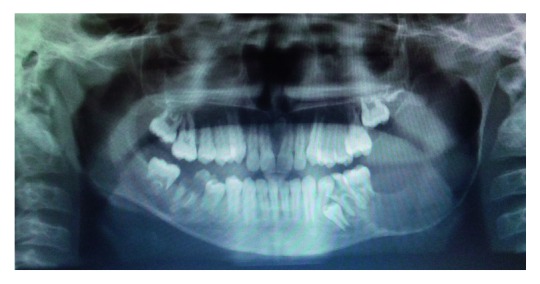
OPG showing large unilateral radiolucencies extending from the 1st permanent molar along the ascending ramus up to the neck of the condyle and involving coronoid process and thinning of the lower border and bowing of angle of mandible.

Radiological examination of orthopantomogram showed the presence of unilocular radiolucencies extending from the last erupted molar to retromolar region (Figure 2).

**Figure 3:**
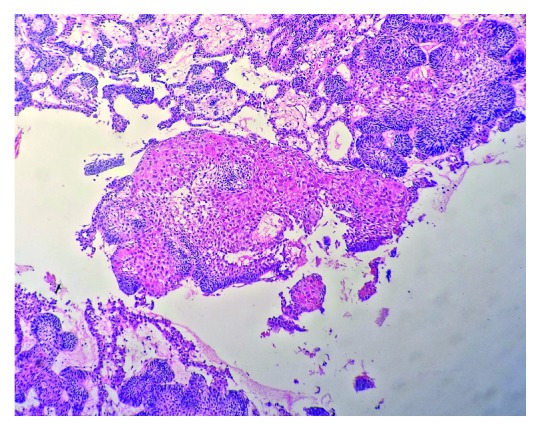
Photomicrograph showing odontogenic epithelial islands arranged in follicles within the connective tissue stroma (H&E 10x view).

**Figure 4:**
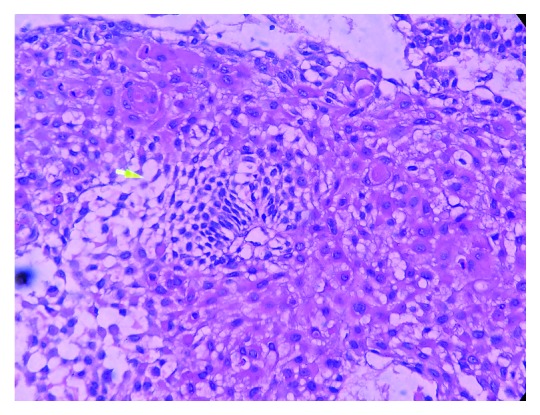
Photomicrograph showing areas of ameloblast-like cells, stellate reticulum-like cells, individual cell keratinisation, and squamous metaplasia.

**Figure 5:**
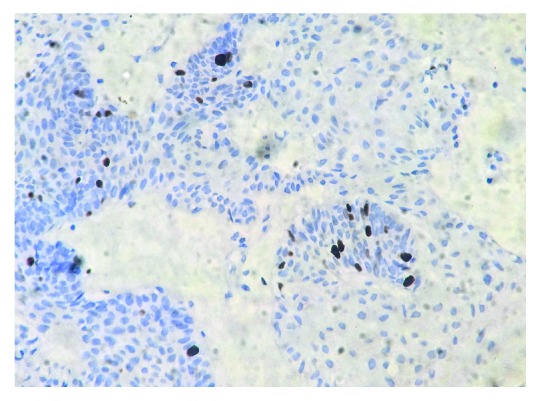
IHC Ki-67 mildly positive in fewer areas.

Based on the above clinical and radiological findings, provisional diagnosis of unicystic ameloblastoma was given. Incisional biopsy was carried out, and the specimen was sent to the Department of Oral Pathology and Microbiology. The gross specimen measured around 1 × 0.6 × 0.4 cm to 0.2 × 0.2 × 0.1 cm in dimension, which was firm in consistency, creamish brown in colour, and irregular in shape. The histopathological examination showed sheets of odontogenic islands lined by tall columnar cells enclosing stellate reticulum-like cells along with squamous metaplasia in the connective tissue stroma extending into the peripheral epithelium. (Figures 3 and 4). These features suggested the diagnosis of acanthomatous ameloblastoma. IHC Ki-67 also showed mild positivity in fewer areas (Figure 5).

**Figure 6:**
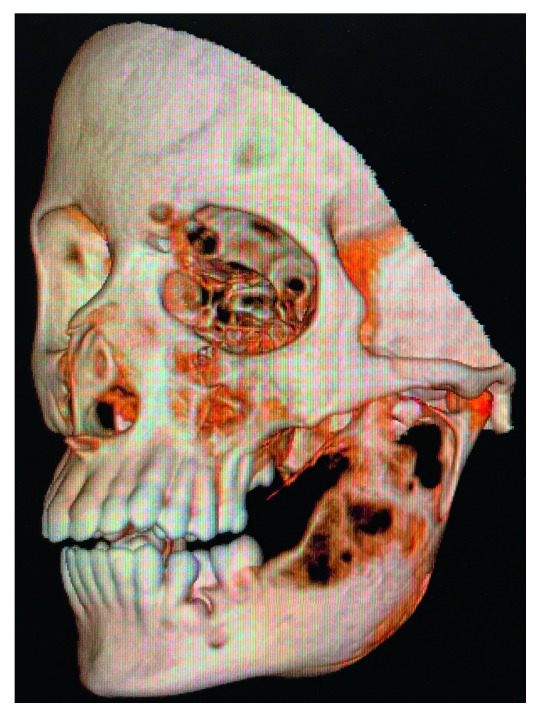
CT scan showing expansile lytic lesion of left ramus of the mandible.

Further radiological investigations proceeded with CT scan neck (plain and contrast) and CT Brain (Figure 6). Serial axial sections of the neck were studied from the nasopharynx up to the thoracic inlet before and after intravenous contrast. The findings revealed that an expansile lytic lesion of size 5.2 × 3.4 × 6.5 cm (AP × Trans × CC) with a largely preserved peripheral cortical rim (with few areas of dehiscence), and no matrix calcification was seen arising from the left mandibular ramus involving the coronoid process but sparing the condyle (superior limit approximately 5 mm from the condyle). On contrast administration, heterogeneous enhancement with multiple nonenhancing foci (likely necrotic areas) was noted. Also, an area of few air pockets was seen within. Serial axial sections of the brain were studied. The findings revealed an expansile lytic lesion arising from the left mandibular ramus, with relative preservation of the surrounding cortical rim (with few areas of dehiscence) and no focal space occupying the lesion. There were no metastatic disseminations evident on these imaging findings which conclude the conventional type of ameloblastoma.

**Figure 7:**
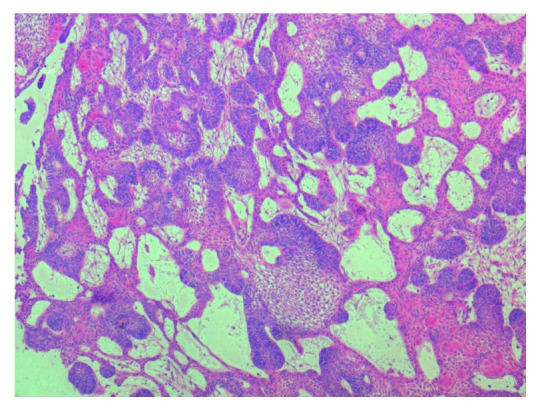
Photomicrograph showing sheets of odontogenic epithelial follicles lined by tall columnar cells enclosing stellate reticulum-like cells within the connective tissue stroma (H&E 4x view).

**Figure 8:**
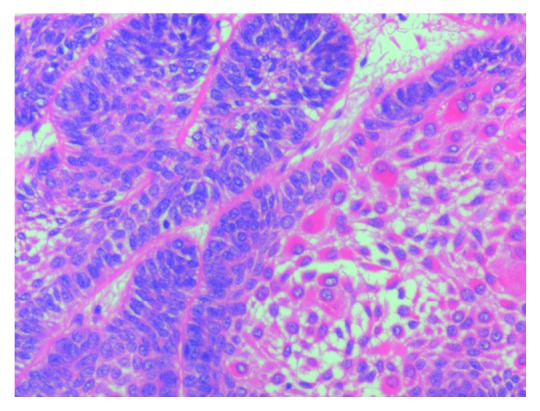
Photomicrograph showing follicles lined by tall columnar cells enclosing stellate reticulum-like cells and squamous metaplasia (H&E 40x view).

**Figure 9:**
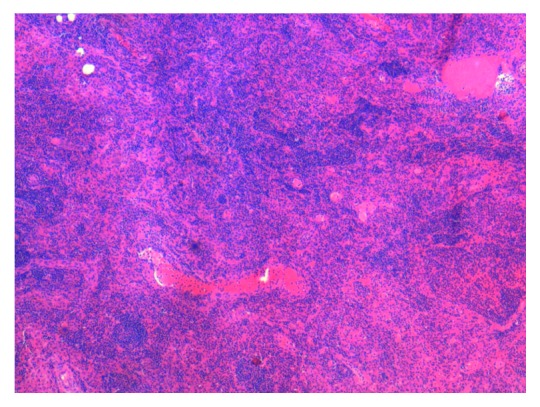
Photomicrograph showing reactive lymph nodes (H&E 20x view).

Surgical treatment was planned under general anaesthesia. Extraction of 74, 34 and hemimandibulectomy of the left mandible was carried out. The resected mandible was reconstructed by free fibular graft. The weight and measurement of the resected specimen was 87 gms and 9 × 6 × 4 cm, respectively. Histopathological examination of the resected specimen revealed anastomosing follicular islands of the odontogenic epithelium lined by tall columnar cells enclosing stellate reticulum-like cells. Squamous metaplasia of the stellate reticulum-like cells was observed. Areas of cystic degeneration of the stellate reticulum-like cells were also seen. A reactive change of lymphoid tissue was seen. Based on the clinical, radiological, and histopathological examination, a final diagnosis of acanthomatous ameloblastoma was made (Figures 7–9). A three-month postoperative follow-up of the patient showed good prognosis with no recurrence.

## 3. Discussion

The occurrence of ameloblastoma is only 1% compared with all oral tumors, and among other Odontogenic tumors, the incidence is 11–18% [3]. Acanthomatous ameloblastoma accounts for the third most common histological variant of ameloblastoma [4]. Occurrence of tumor is more commonly seen on the mandible than the maxilla [5].

Involvement of the mandibular ramus region is seen in 80% of cases as similar to our case. These lesions are commonly encountered in the 3rd to 5th decades of life, which was not consistent with our case as our patient was in the 2nd decade of his life.

Though ameloblastoma is a benign tumor in nature, its clinical presentation shows a painless, slow-growing mass, with displacement of teeth, malocclusion, loose teeth along with local aggressiveness and frequent invasion into the alveolar bone [6, 7]. Similar clinical features were observed in our case which had a severe local aggressiveness and invasion of the alveolar bone.

Radiological findings of ameloblastoma usually show expansile, unilocular/multilocular radiolucencies and typical ‘soap bubble appearance'. In our case, the OPG revealed expansile lytic lesion along with thinning of cortical plates.

Histopathological examination of acanthomatous ameloblastoma shows squamous metaplasia of stellate reticulum and the formation of keratin within the tumor islands [8]. The characteristic histopathological features of AA were well appreciated in the histopathology of our case.

Dentigerous cyst and odontogenic keratocyst may possibly be the clinical differential diagnosis considering the age, site, size, and extent of the swelling.

Radiological differential diagnosis may include odontogenic myxoma and central giant-cell granuloma.

Payne et al. [2] suggested that extensive surgical resection and reconstruction can greatly affect the growth of the craniofacial skeletal region in the paediatric population. In our case, we have performed hemimandibulectomy followed by reconstruction with free fibular graft to overcome the functional and aesthetic disturbances.

Assessment of cellular proliferative activity of the odontogenic cysts and tumors is mainly done by the Ki-67 index which plays an important prognostic marker of tumor recurrence, biological behaviour, and local invasiveness. The peripheral cell layer of the epithelial islands when compared to central cells usually shows Ki-67 positivity, which suggests that peripheral cells are more proliferative [9].

Immunohistochemical analysis of Ki-67 labelling has a brownish staining of nuclear areas as well as focal and total nuclear positivity of tumor cells [10] similar to our IHC findings of Ki-67 on an incisional biopsy specimen.

## 4. Conclusion

Our case report has various significant features which discerned it from previously reported cases of acanthomatous ameloblastoma. The case being discussed is presented with a diffuse swelling involving complete ramus of the mandible in a 12-year-old boy. To avoid higher chances of recurrence and to lessen the aggressive, rapidly growing and lytic nature of acanthomatous ameloblastoma, radical surgical treatment has been performed in our patient.
